# Overexpression of FERM Domain Containing Kindlin 2 (FERMT2) in Fibroblasts Correlates with EMT and Immunosuppression in Gastric Cancer

**DOI:** 10.1155/2024/4123737

**Published:** 2024-02-06

**Authors:** Sheng-yan Yin, Yuan-jie Liu, Jie-pin Li, Jian Liu

**Affiliations:** ^1^The Second Affiliated Hospital of Nanjing University of Chinese Medicine, Jiangsu Second Chinese Medicine Hospital, Nanjing, Jiangsu 210029, China; ^2^Affiliated Hospital of Nanjing University of Chinese Medicine, Jiangsu Province Hospital of Chinese Medicine, Nanjing, Jiangsu 210029, China; ^3^No. 1 Clinical Medical College, Nanjing University of Chinese Medicine, Nanjing, Jiangsu 210023, China

## Abstract

The mesenchymal feature, dominated by epithelial mesenchymal transition (EMT) and stromal cell activation, is one of the main reasons for the aggressive nature of tumors, yet it remains poorly understood. In gastric cancer (GC), the fermitin family homolog-2 (*FERMT2*) is involved in macrophage signaling, promoting migration and invasion. However, the function of *FERMT2* in fibroblasts remains unclear. Here, we demonstrated that downregulation of *FERMT2* expression can block EMT in GC cells by inhibiting fibroblast activation in vitro. Furthermore, we found that, in addition to the known pathways, fibroblast-derived *FERMT2* promotes M2-like macrophage growth and that in human GC samples, there is a strong positive correlation between FERMT2 and CD163 and CD206 levels. Notably, high *FERMT2* expression was significantly associated with poor clinical outcomes and was upregulated in patients with advanced disease. Taken together, our results provide evidence that the fibroblast-FERMT2-EMT-M2 macrophage axis plays a critical role in the GC mesenchymal phenotype and may be a promising target for the treatment of advanced GC.

## 1. Introduction

Gastric cancer (GC), one of the most lethal human diseases globally, imparts a heavy financial burden on the Chinese health care system [1]. According to Global Cancer Observatory (GLOBOCAN) data, there are more than one million newly diagnosed cases of GC every year, making it the fifth leading cause of cancer-related deaths in the world [2]. The global population is now an aging one, and the incidence of GC is expected to increase. As we all know, the early diagnosis rate, radical resection rate, and 5-year survival rate of patients with GC are low [3]. Although surgical techniques and adjuvant therapy have made great progress, the prognosis for patients with advanced-stage GC is still discouraging [4, 5]. Currently, the prognosis of GC patients depends on the degree of progression of metastatic disease, the sensitivity of patients to drug therapy, and whether the patient can tolerate the full course of treatment [6]. Human epidermal growth factor receptor 2 (HER2) is activated by amplification or overexpression in GC. In advanced HER2-positive GC, cytotoxic chemotherapy (CCT) in combination with agents targeting HER2 is effective in improving survival [7, 8]. HER2-targeted therapies include the monoclonal antibody trastuzumab and the small-molecule kinase inhibitor lapatinib [9]. In HER2-negative gastric cancer patients, the combination of immune checkpoint inhibitors (ICIs) and CCT has been shown to limit tumor cell repopulation and optimize the outcome of both treatments [10, 11]. Thus, while targeted therapies and immunotherapy offer patients new treatment options, chemotherapy continues to play an integral role alone or in combination with other regimens. It is a challenge for scholars and clinicians to identify new and reliable therapeutic targets for the individualized treatment and prognosis prediction of GC patients [12].

With a deeper understanding of cancer biology, cancer progression is no longer considered an isolated event of the accumulation of mutations and associated malignant features in the primary tumor [13]. Tumor cell-derived cytokines alter the biological composition of the stroma by affecting nonparenchymal cells to induce angiogenesis and promote cancer-associated fibroblast (CAF) activation [14, 15]. Stromal components lead to signals from the tumor microenvironment (TME) to support tumor progression, and TME is highly immunosuppressive as it crosstalks with tumor cells to educate inflammatory cells [16, 17]. Of all the types of nonparenchymal cells with different functions, fibroblasts and macrophages are highly enriched in TME, and their overall function favors the survival and migration of cancer cells [18–20]. Therefore, TME studies of macrophages and fibroblasts can provide new insights into the malignant behavior of GC.

The epithelial-mesenchymal transition (EMT) process is a driver of epithelial tumor spread as a transdifferentiation process that reprograms epithelial cells into a mesenchymal-like phenotype [21, 22]. Although many transcription factors have been identified to activate EMT, the complete reprogramming process is mainly triggered by three groups of transcription factors, including the E homeobox-binding (ZEB), Snail, and Twist families [23, 24]. These transcriptional activators are upregulated in tumor tissue, conferring greater aggressiveness to tumor cells and correlating with a poor prognosis in cancer patients [25, 26]. On the other hand, EMT contributes to the maintenance of cancer cell stemness [27], the latter leading to cancer cells entering a quiescent state and possessing a high resistance to treatment [28]. Importantly, there is a significant overlap between the EMT signal and the fibroblast activation signal [29], and indeed, fibroblasts release a variety of signals to tumor cells to help them complete the EMT process [30]. Thus, mesenchymal signaling has complex implications for tumors.

The kindlin or fermitin (FERMT) family is a group of adapter or scaffold proteins with names defined by the 4.1 protein, ezrin, radixin, and moesin (FERM) structural domains that mediate transmembrane integrin adhesion receptor signaling by acting in concert with talin [31, 32]. FERMTs comprise three members: *FERMT1* is restrictedly expressed on epithelial cells, *FERMT2* is widely expressed, and FERMT3 is predominantly located in the hemopoietic system [33, 34]. *FERMT2* has been found to be implicated in tumor pathogenesis and is highly expressed in a variety of cancers, including breast and colorectal cancers [35, 36]. Although *FERMT2* shows a clear association with macrophages in GC, the relationship between *FERMT2* and the mesenchymal phenotype, particularly fibroblasts, remains incompletely elucidated.

In the current study, we assessed the expression and prognostic significance of *FERMT2* at the pancancer level. Our data suggested that downregulation of *FERMT2* expression inhibited fibroblast activation and affected the EMT process in GC cells in a fibroblast-dependent manner. Furthermore, fibroblast-derived *FERMT2* was determined to upregulate the expression of M2 macrophage markers. Importantly, we also demonstrated that *FERMT2* was involved in the immune exclusion of GC and that patients with high variability of *FERMT2* had difficulty benefiting from immunotherapy. Overall, we established a fibroblast-FERMT2-EMT-M2 macrophage axis, which may play a key role in the mesenchymal phenotype of GC and serve as a promising target for advanced cancer therapy.

## 2. Materials and Methods

### 2.1. Reagents

Detailed information on all reagents and antibodies used in this study is provided in Supplementary material (Table S1). As recommended by manufacturers or based on previous studies, the antibody concentrations used are those used in previous studies.

### 2.2. Public Datasets

Through the University of California, Santa Cruz (UCSC) web browser, 375 GC samples were downloaded, including gene expression profiles, clinical data, and mutation data [37]. The Cancer Genome Atlas Stomach Adenocarcinoma (TCGA-STAD) gene expression profiles (FPKM: fragments per kilobase of transcript per million values) were transformed into transcripts per kilobase million (TPM) using R software. To further analyze the gene expression data, R was used to construct the data matrix.

The single-cell dataset GSE167297 (expression matrix) was obtained from the TISCH portal (http://tisch.comp-genomics.org/), and metainformation provided by the original authors was used.

### 2.3. Preparation of Single-Cell Suspensions from Surgical Specimens

The GC specimens' molecular characteristics and cell populations were assessed via scRNA-seq. Specimen collection was approved by the Ethics Committee of the Jiangsu Second Chinese Medicine Hospital (approval number: 2022SEZ-022). The two participants in this study did not receive chemotherapy or radiation therapy prior to surgery. The samples obtained through surgical means were immediately processed. The biopsied samples were divided into small fragments using an iris scissor. These fragments were then immersed in a digesting solution containing phosphate-buffered saline (PBS) and incubated for 30 minutes at a speed of 800 rpm and a temperature of 37°C. After that, the fragments were subjected to a 1-hour incubation at 37°C with collagenase II, trypsin, and DNase. The suspension that was made was thinned out with 4 mL of Dulbecco's modified Eagle's medium (DMEM), put through a 40-micron cell mesh filter, and then spun at 250 g for 5 minutes. The cell pellet was washed twice with PBS and then suspended in a solution that lysed red blood cells. It was then incubated at a temperature of 4°C for 10 minutes. After that, it was combined with 10 mL of cold PBS and centrifuged again at a speed of 250 g for 10 minutes. The obtained pellet was dissolved in 5 mL of a PBS solution lacking calcium or magnesium. Additionally, bovine serum albumin (BSA) at a concentration of 0.04% weight/volume was added. Subsequently, 10 *μ*L of the cell suspension was measured using a hemocytometer under an inverted microscope. Live cells were successfully identified using trypan blue.

### 2.4. 10X Genomics scRNA-seq and Data Processing

By using the CellRanger package (version 3.1), 10X Genomics sequencing data were aligned and measured against a human reference genome (hg19) [38]. Cells with library size < 200, mitochondrial transcript ratio > 0.4, and gene expression < 3 were removed. The remaining 8378 cells' gene expression matrix was normalized and then adjusted by regressing the total cellular UMI counts. Highly variable genes were calculated using the “FindVariableGenes” tool, which was used to specify the quantile-normalized variance > 0.5 and the mean expression between 0.125 and 5. Subsequently, a uniform manifold approximation and projection (UMAP) dimensional reduction was performed by selecting 20 crucial principal components (PCs) based on principal component analysis (PCA). The “FindClusters” function was used to cluster all cells, resulting in 20 different clusters. These clusters were then annotated into 4 main cell types using a combination of manual and automatic annotation with the help of singleR. Further, in order to simulate the dynamic evolution of fibroblasts, the “monocle3” package was conducted for cellular trajectory reconstruction analysis using gene counts and expression. The gene expression patterns of *FERMT2* and well-known fibroblast activation markers (actin alpha 2 (*ACTA2*), caveolin 1 (*CAV1*), fibroblast activation protein alpha (*FAP*), integrin subunit beta 1 (*ITGB1*), and tenascin C (*TNC*)) were detected along the pseudotime using the “plot_genes_in_pseudotime” function.

In addition, the processing with respect to publicly available data GSE167297 was similar to that described above.

### 2.5. Enrichment Analysis

Differentially expressed genes (DEGs) based on *FERMT2* median expression level in TCGA-STAD were obtained by “Limma” package [39]. An absolute log2 fold change (FC) > 2 and an adjusted *P* value of 0.05 were considered to be statistically significant and were used in subsequent analysis. Gene Ontology (GO) and hallmark enrichment analysis were conducted using the “clusterProfiler” package [40, 41]. We considered enrichment to be significant if the *P* > 0.05 value was determined.

### 2.6. Construction of Coexpression Networks

We used a query for individual protein names (“FERMT2”) to search the GeneMANIA website (http://www.genemania.org), which includes 660,443,499 interactions from nine organisms. Thus, a network of FERMT2-related proteins based on physical interactions and coexpression was obtained.

### 2.7. Expression and Survival Curve Analysis

With the “survival” R package, we were able to visualize the relationship between the RNA sequences and the clinical characteristics of TCGA pancancer. We also used the GEPIA2 “survival graph” module to obtain OS (overall survival) significance graph data for *FERMT2* in all TCGA tumors [42]. For the Kaplan-Meier (KM) curves plotting, low- and high-expression cohorts were further separated based on optimal cut-off values (the minimum *P* value) to obtain the most statistically significant results. Optimal group cut-off values were obtained by the “surv_cutpoint” function from the “survminer” package.

### 2.8. Immune Analysis

Using single-sample gene set enrichment analysis (ssGSEA), we quantified the level of immune cell infiltration in GC, and we calculated a tumor immune dysfunction and exclusion (TIDE) score that predicted the potential response to potential immune checkpoint blockade (ICB) [43]. For pairwise correlation comparisons, the Spearman rank correlation coefficients and *P* values were calculated (*P* < 0.05 was considered statistically significant).

### 2.9. Estimation of Stroma Score in Pancancer

“Estimate” algorithm was used to calculate the stroma score for each sample in the pancancer dataset to assess the degree of fibrosis [44]. Correlations between stroma score and *FERMT2* were then calculated according to Spearman's method.

### 2.10. Roles of *FERMT2* in the Normal Human Tissues

In this study, the expression profile of *FERMT2* in normal human tissues was visualized using the single-cell type section in the Human Protein Atlas (HPA) database (https://www.proteinatlas.org/), which contains scRNA-seq data based on 29 individual tissues and peripheral blood mononuclear cells (PBMCs).

### 2.11. Roles of FERMT2 in the Pancancer Microenvironment

Tumor Immune Estimation Resource (TIMER) [40] was used to evaluate the relationship between *FERMT2* and fibroblast in pancancer. In addition, the STOmicsDB online tool (https://db.cngb.org/) based on spatially resolved transcriptome data was used to analyze the spatial expression levels and overlap of *FERMT2* and the fibroblast markers *ACTA2* and decorin (*DCN*) in cervical cancer (CESC), prostate adenocarcinoma (PRAD), breast invasive carcinoma (BRCA), glioblastoma multiforme (GBM), ovarian serous (OV), and renal cell carcinoma (RCC). At single-cell resolution, TISCH was used, which contains scRNA-seq data from Gene Expression Omnibus (GEO) and ArrayExpress across human tissues. Data for 2045746 cells from 79 datasets were processed uniformly and were able to elucidate the components of the TME at the level of annotated clusters. In this study, we used the TISCH dataset to reveal the expression of *FERMT* between various cell subtypes at the pancancer level.

### 2.12. Inclusion/Exclusion Criteria for Participants and Sample Collection

The retrospective study involved a cohort of 56 patients who underwent treatment for GC at the Jiangsu Second Chinese Medicine Hospital over the period from May 1, 2022, to May 1, 2023. Specimens obtained from patients were collected and stored following the approved protocols. Ultimately, 16 individuals were omitted from the study due to their failure to meet the inclusion criteria. The final cohort consisted of 22 males and 18 females, with an average age of 59. The classification of tumor grade and stage was determined based on the criteria provided by the International Union Against Cancer. There were 12 in stage III and 28 in stage IV. Anonymization and deidentification of all patient records were conducted before analysis. As soon as samples were obtained, they were frozen and immediately stored in liquid nitrogen. Inclusion criteria are as follows: (1) All patients diagnosed with GC for the first time had clinical examination, gastroscopy, magnetic resonance imaging (MRI), positron emission tomography (PET)-computed tomography (CT)/CT, and hematological diagnostics. All patient's pathology samples were saved for independent validation by two different specialists. (2) Complete clinical data were available for every patient. We evaluated the efficacy of those patients who were receiving traditional chemotherapy (SOX: S-1 plus oxaliplatin or XELOX: capecitabine plus oxaliplatin) in combination with nivolumab (360 mg intravenously every 3 weeks). A PET-CT or CT scan was conducted every 6 weeks or as required by the patient's clinical condition to assess their radiologic status. Evaluation Criteria in Solid Tumors, v1.1 [45], was the basis for tumor response evaluation. The study excluded the following patients: (1) those with a pathological diagnosis other than GC, such as a gastric stromal tumor; (2) patients who died during treatment; (3) patients with incomplete data or who were lost to follow-up; (4) patients with an infection, immune system disorder, or blood system condition; (5) patients who underwent local radiotherapy or radiofrequency ablation; (6) patients who were HIV positive; and (7) patients who were unable to tolerate adverse reactions.

### 2.13. Immunohistochemical (IHC) and Multiple Immunofluorescence (mIF) Staining

mIF staining was performed on GC paraffin sections to validate the coexpression of FERMT2 and fibroblast markers (FAP and ACTA2) and the immune exclusion potential of FERMT2. IHC staining was performed on GC paraffin sections to measure the correlation between FERMT2 and M2 macrophage classical markers (CD163 and CD206) and to explore the significance of FERMT2 for immunotherapy. Specific experimental procedures as well as H-sore procedures were carried out as previously described [46, 47].

### 2.14. Cell Culture

Chinese Academy of Sciences' 113-cell repository provided the undifferentiated human gastric cancer cell line HGC-27 and the human monocytic cell THP-1 (Shanghai, China). The American Type Culture Collection (ATCC) provided the human gastric cancer cell line AGS. Procell Life Science and Technology Co. Ltd. provided human gastric cancer tissue-derived fibroblasts (CAFs). The RPMI-1640 medium was used to culture HGC-27, AGS, and THP-1, along with 10% fetal bovine serum (FBS). CAFs were cultured in DMEM with 10% FBS. Every cell was cultured in an environment with 5% CO_2_ at 37°C [48].

### 2.15. RNAi Plasmid Construction and Transfection

GeneChem (Shanghai, China) created all of the plasmids described below. We chose the best inhibitory efficiency from three short hairpin interfering RNA targeting *FERMT2* (5′-GAATCAATCAGCTTTACGA-3′) for the following investigations. The *si-FERMT2* and the nontargeting control (NC) sequence plasmid (5′-CCTAAGGTTAAGTCGCCCTCG-3′) were transfected into 70% confluent cells using Lipofectamine 3000 per provided protocols.

### 2.16. Colony Formation Assays in Coculture Unit

Briefly, GC cells were placed in the bottom chamber of a 6-well plate and cultivated for approximately 14 days (top chamber: CAF cells 500, bottom chamber: GC cells 500). After the application of 0.5% crystal violet (CV) for 10 minutes at a temperature range of 20–25°C, the colonies were stained and subsequently counted using compound light microscopy (Olympus BX53, Japan).

### 2.17. Transwell Assay in Coculture Unit

30,000 GC cells were put in the upper chambers of 24-well Transwell plates with 200 *μ*L of DMEM without FBS. The bottom chambers have been filled with 500 *μ*L of medium containing 10% FBS and 30,000 CAFs. Matrigel was applied to the Transwell chambers. Following a 24-hour period of coculture, the top chambers of the Transwell were rinsed with 1% PBS. Following a 15-minute immersion in a 4% paraformaldehyde solution, the chambers underwent staining with a 0.1% solution of CV at a temperature range of 20–25°C. A light microscope (Olympus BX53, Japan) was employed to acquire images of the cells that had migrated into the lower chambers. The software ImageJ was then applied to calculate the total number of cells present in the photographs.

### 2.18. Western Blotting (WB)

In this investigation, the WB was constructed using the methodology described in a prior publication [49]. The cells were lysed in radioimmunoprecipitation assay buffer (RIPA buffer), and the protein content was quantified using the Bradford assay [50]. 20 *μ*g samples were added to SDS-polyacrylamide gel electrophoresis (SDS-PAGE) using 10%–8% gel. The transfer of membrane proteins using polyvinylidene fluoride (PVDF) was incubated for 30 minutes with the addition of 5% bovine serum albumin (BSA) at 20–25°C. The blots were subsequently incubated using appropriate primary antibodies overnight at 4°C. Following three rinses in a Tris-buffered saline solution containing 0.05% Tween 20, the blots were exposed to the secondary antibodies. The protein *β*-actin functioned as a reference.

### 2.19. Wound Healing Assay in Coculture Unit

The migratory capacity of GC cells was assessed by wound healing tests conducted in the coculture unit. The cells were cultured to reach full coverage in medium without serum in 6-well plates for 24 hours, using a cell concentration of 4 × 10^5^ GC in the bottom chamber of each well. After the medium was removed, the cell layer was scraped using a 10 *μ*L pipette tip. The assessment of wound healing was conducted at 0, 12, and 24 hours using a light microscope (Olympus BX53, Japan) with a magnification of approximately 200x.

### 2.20. Xenograft Tumor Model

The ethics committee at the Jiangsu Province Hospital of Traditional Chinese Medicine authorized animal experiments (approval number: 2022DW-72-02), which were done in accordance with the “Guide for the Care and Use of Laboratory Animals”. Male nude BALB/c mice, aged 4 weeks, were acquired from the Beijing Institute of Biomedicine (Beijing, China) (Certificate No. SYXK2019-0010). CAFs transfected with si-*FERMT2*/NC (5 × 10^5^) and AGS (5 × 10^5^) were subcutaneously injected into the right armpit of mice at a dose of 1 × 10^6^ cell/mouse [51]. The presence of tumors was discovered after seven days. Following that, the maximum and minimum tumor sizes were measured twice weekly. The mice were CO_2_ euthanized 35 days after inoculation, and the tumors were removed. The euthanasia was performed in accordance with the American Veterinary Medical Association (AVMA's) Guidelines for Humane Animal Euthanasia [52]. The volume of the tumor was determined using the formula *V* = 1/2*ab*^2^, and growth curves for the tumor were drawn.

### 2.21. Differentiation of Monocytes into Macrophages Was Induced with Propylene Glycol Methyl Ether Acetate (PMA)

The THP-1 cell is a type of human leukemia cell-derived monocyte line that can develop similar features to primary macrophages when stimulated by PMA, both in terms of phenotypic and functional characteristics [53, 54]. THP-1 cells were centrifuged and resuspended in RPMI-1640 medium; after 48 hours of induction with 10 ng/mL PMA, 95% of THP-1 cells transformed from suspension growth to wall growth and from round to irregular shape and increased in size, cell pulp was loosened, cell nucleus enlarged obviously, a large number of obvious organelles were visible, and a small amount of protrusion around the cytosol was visible (Figure S1).

### 2.22. Establishment of a Coculture Unit

GC cells and CAFs were cultivated together in a noncontact coculture system utilizing the Transwell method. The culture medium was renewed every 48 hours [55]. In the subsequent examinations, the CAFs and GC cells were allocated to either the top or lower chamber based on the specific requirements of the experiment.

## 3. Results

### 3.1. The Expression of *FERMT2* in Pancancer

**Figure 1:**
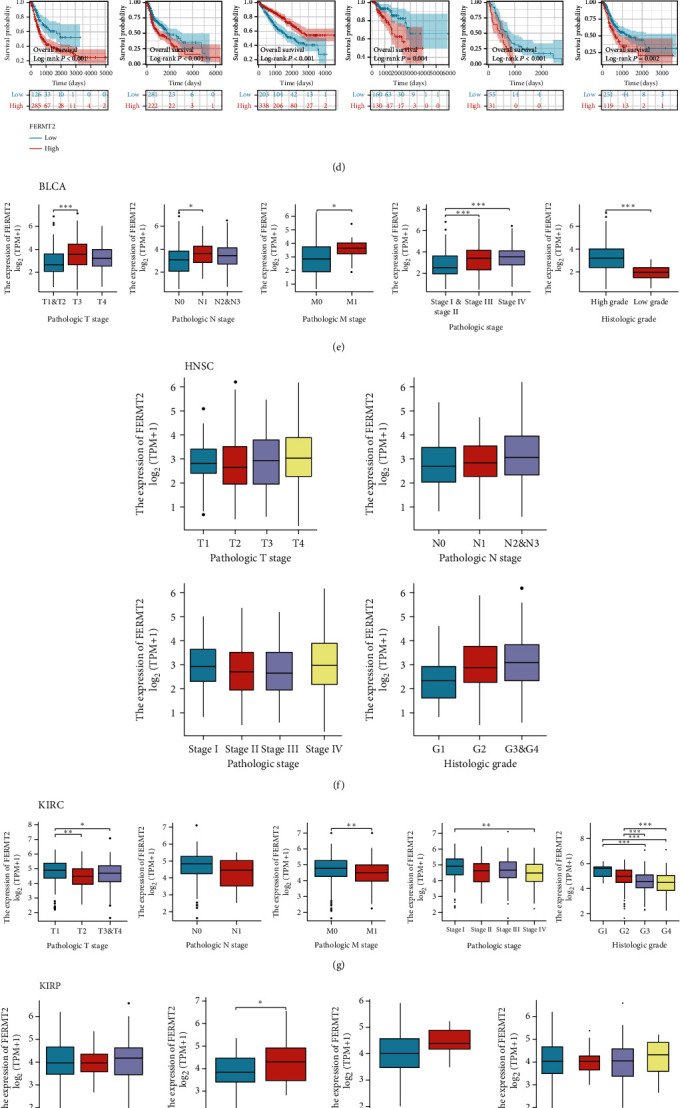
Analysis of FEERMT2 expression and prognosis in pancancer. (a) FERMT2 expression in cancers in The Cancer Genome Atlas (TCGA) paired samples. The Kruskal-Wallis test was conducted. (b) FERMT2 expression in breast invasive carcinoma (BRCA), colon adenocarcinoma (COAD), kidney renal clear cell carcinoma (KIRC), liver hepatocellular carcinoma (LIHC), lung adenocarcinoma (LUAD), skin cutaneous melanoma (SKCM), thyroid carcinoma (THCA), pancreatic adenocarcinoma (PAAD), and stomach adenocarcinoma (STAD) from the Human Protein Atlas (HPA) database. GEPIA2 tool was conducted to perform overall survival analyses of different tumors in TCGA by FERMT2 gene expression (c, d). The survival map and Kaplan-Meier curves with positive results are shown, and the optimal cut-off value to be used for sample grouping is derived from the “surv_cutpoint” function in the “survminer” package (d). Based on the TCGA data, the expression levels of the FERMT2 gene were analyzed by the main pathological parameters of (e) bladder urothelial carcinoma (BLCA), (f) head and neck cancer (HNSC), (g) KIRC, (h) kidney renal papillary cell carcinoma (KIRP), (i) mesothelioma (MESO), and (j) STAD. Log2 (TPM+1) was applied for log scale. ^∗^*P* < 0.05, ^∗∗^*P* < 0.01, and ^∗∗∗^*P* < 0.001.

Paired samples from the TCGA database were used to analyze the expression of *FERMT2* in cancerous and normal tissues. The results showed that *FERMT2* was downregulated in most cancer types compared to normal tissues, including bladder urothelial carcinoma (BLCA), breast invasive carcinoma (BRCA), colon adenocarcinoma (COAD), head and neck cancer (HNSC), kidney chromophobe (KICH), kidney renal clear cell carcinoma (KIRC), kidney renal papillary cell carcinoma (KIRP), liver hepatocellular carcinoma (LIHC), lung adenocarcinoma (LUAD), lung squamous cell carcinoma (LUSC), PRAD, rectum adenocarcinoma (READ), thyroid carcinoma (THCA), and uterine corpus endometrial carcinoma (UCEC) (Figure 1(a), *P* < 0.05). IHC showed representative staining of cancer and normal tissues and showed that FERMT2 was expressed in the nucleus and cytoplasm (Figure 1(b)). We then divided the pancancer cases into high- and low-expression groups according to the optimal cut-off values for *FERMT2* expression levels, using mainly data from TCGA, to investigate the correlation between *FERMT2* expression and the prognosis of patients with different tumors. As shown in Figures 1(c) and 1(d), high *FERMT2* expression predicted poor prognosis of overall survival (OS) for patients with BLCA (*P* < 0.001), HNSC (*P* < 0.001), KIRP (*P* = 0.004), mesothelioma (MESO) (*P* < 0.001), and STAD (*P* = 0.002), while the opposite was true for KIRC (*P* < 0.001). We also found that *FERMT2* transcriptome levels correlated with clinicopathological stage of BLCA, KIRC, and STAD in the six prognostically significant cancer types described above (Figures 1(e)–1(j)).

### 3.2. Identification of FERMT2 Association with Fibroblasts and Stroma

**Figure 2:**
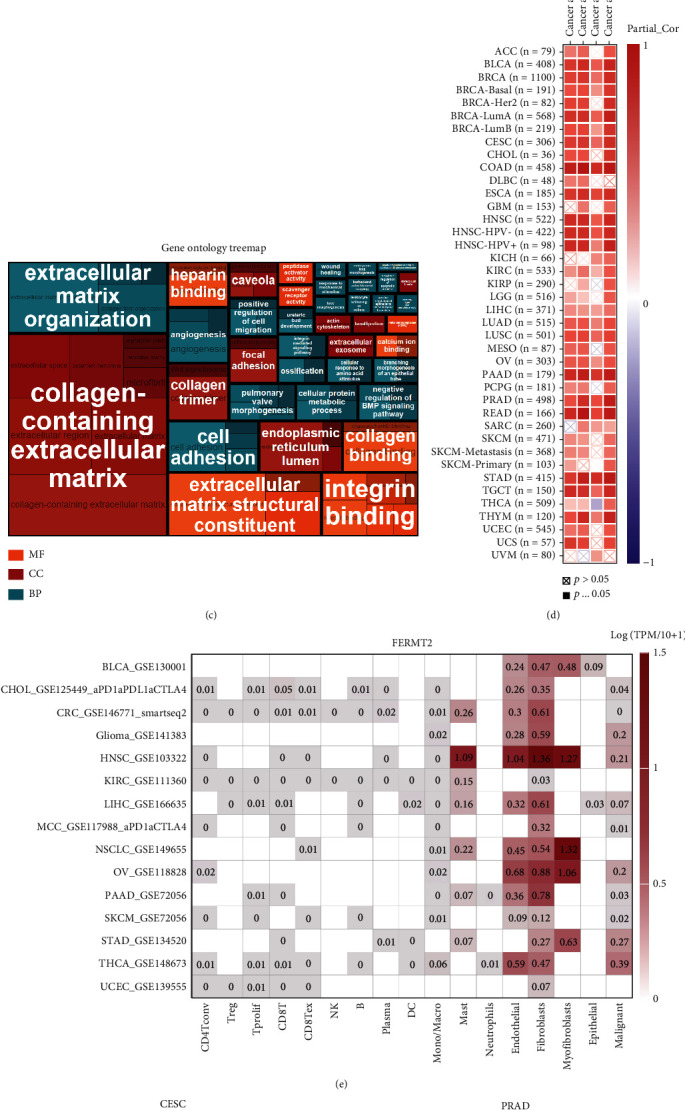
FERMT2 is a biomarker of fibroblast infiltration in normal and pancancer tissues. (a) Uniform manifold approximation and projection (UMAP) plot showing FERMT2 expression in normal single-cell clusters (HPA database). (b) Heatmap showing gene expression in FERMT2-specific cluster. *x*-axis shows sample types across 30 tissues, and *y*-axis shows genes in selected clusters. The vertical line on the left indicates the confidence level of the gene assigned to the cluster. (c) The Gene Ontology (GO) tree shows the summary results of the GO term overexpression analysis. The most prominent terms are summarized as a single box with white text. Orange, red, and blue encode different GO term domains, respectively. (d) The heatmap of associations between FERMT2 level and fibroblast infiltration in pancancer was calculated by four algorithms on Tumor Immune Estimation Resource (TIMER) web tool. (e) FERMT2 expression in cancer single-cell clusters obtained from TISCH online tool. Spatial transcription sections showing the spatial expression of *FERMT2*, *ACTA2*, and *DCN* fibroblast marker in (f) cervical cancer (CESC), (g) prostate cancer (PRAD), (h) breast cancer (BRCA), (i) glioblastoma (GBM), (j) ovarian cancer (OV), and (k) kidney cell carcinoma (RCC). The dot color represents the expression level of the selected gene. Colocalization was highlighted by the red box. (l) Correlation between FERMT2 and tumor stroma scores assessed by the “Estimate” algorithm in pancancer. Spearman's method was used.

Here, we attempted to characterize the expression pattern of *FERMT2* at a higher resolution. Single-cell data across 30 sample types in Figure 2(a) indicated that *FERMT2* was expressed restrictively on fibroblasts (orange marker, HPA database).  Figure 2(b) illustrates the differential expression of feature genes of this cell cluster in different tissues. Enrichment analysis indicated that this fibroblast cluster was closely associated with the collagen matrix-associated pathway (Figure 2(c)). For cancer-associated fibroblasts, we performed multiple algorithms on TIMER to analyze the correlation between their infiltration levels and *FERMT2* expression in pancancer, and a significant positive correlation was observed in almost all tumor types (Figure 2(d)). Further, we retrieved *FERMT2* expression from TISCH in tumor single-cell data. As shown in Figure 2(e), *FERMT2* was expressed by fibroblasts or myofibroblasts in BLCA, CHOL, colorectal cancer (CRC), glioma, HNSC, KIRC, LIHC, Merkel cell carcinoma (MCC), non-small-cell lung cancer (NSCLC), OV, pancreatic adenocarcinoma (PAAD), skin cutaneous melanoma (SKCM), STAD, THCA, and UCEC. In addition, spatial transcriptional data on STOmicsDB were obtained to describe the spatial overlap of *FERMT2* and the fibroblast biomarkers *ACTA2* and *DCN* in CESC, PRAD, BRCA, GBM, OV, and RCC cancer tissues (Figures 2(f)–2(k)), and as expected, *FERMT2*, *ACTA2*, and *DCN* showed similar spatial distributions, implying a potentially strong association of *FERMT2* with fibroblasts. Fibroblasts have been widely shown to induce extracellular matrix deposition, the latter leading to tumor-associated fibrosis and predicting an unfavorable prognosis [56, 57]. Now that the above results have demonstrated the strong association between *FERMT2* and fibroblasts, we calculated the correlation between stroma scores and *FERMT2* in 30 solid tumors based on the “Estimate” method and, as expected, the two were significantly positively correlated in most tumor types (Figure 2(l), except sarcoma (SARC), adrenocortical carcinoma (ACC), THCA, uveal melanoma (UVM), SKCM, KIRP, and KICH).

### 3.3. Characterization of FERMT2 as a Marker of Fibroblast Activation

**Figure 3:**
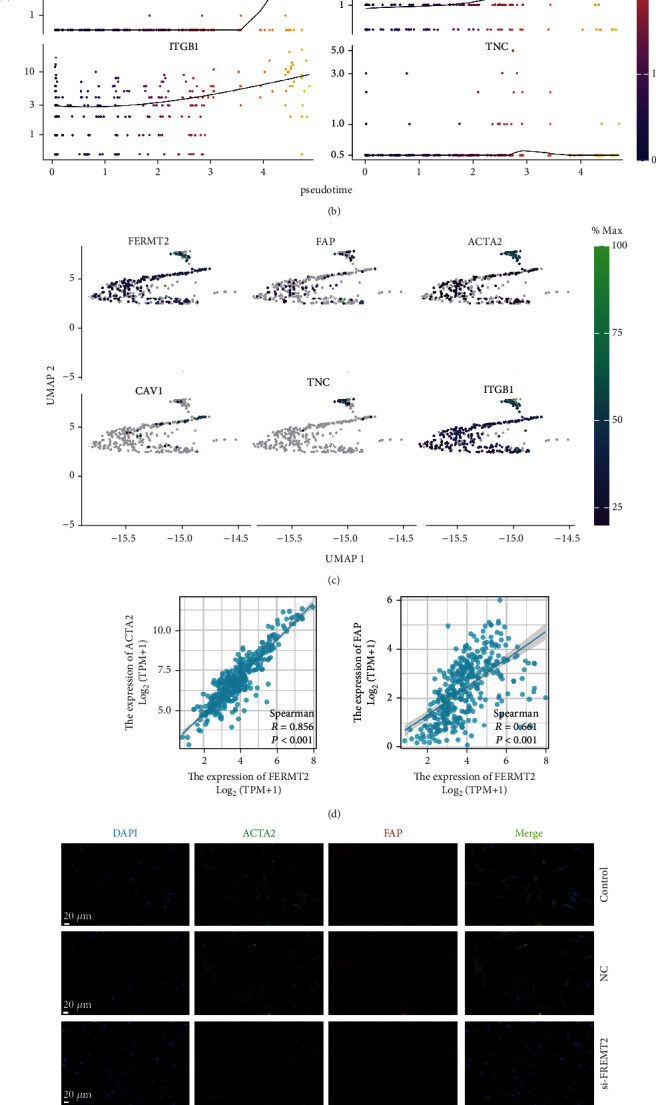
Analysis of the relationship between FERMT2 and fibroblast activation. (a) Pseudotime trajectory of all the fibroblasts from cluster 8 to cluster 19. All the fibroblasts were colored by their assigned pseudotime values. (b) Jitter plots showing the expression level of the fibroblast activation markers and *FERMT2* changing with pseudotime. (c) UMAP dimensionality reduction visualizes similarity of expression profiles of *FERMT2* and fibroblast activation markers. (d) Correlation of the *FERMT2* expression levels with fibroblast activation markers (*ACTA2* and *FAP*) mRNA levels in GC tissues based on TCGA-STAD (Spearman method, *n* = 375). (e, f) Interfering with FERMT2 expression in vitro inhibits fibroblast activation (scale bars = 20 *μ*m). One-way ANOVA was conducted. Asterisk represents *P* value (^∗∗^*P* < 0.01, ^∗∗∗∗^*P* < 0.0001). Data are representative of three experiments (mean ± SEM). (g) Representative pictures of pathological hematoxylin and eosin (HE) staining of FERMT2 high- and low-expression samples (scale bars, 100 *μ*m and 20 *μ*m enlarged images). (h) Multiplex immunofluorescence staining images of FERMT2, ACTA2, and FAP in the GC tissue. The representative view of the costaining of FERMT2, ACTA2, and FAP is shown in the enlarged images view below. Scale bars, 100 *μ*m and 20 *μ*m enlarged images. Nuclei (DAPI) in blue.

The full application of single-cell analysis techniques will allow us to more easily understand the microenvironment composition in GC. Here, we sought to explore the expression patterns of *FERMT2* at the single-cell level. In GC samples containing 8762 cells, we observed that *FERMT2* was highly enriched in fibroblasts, consistent with the expression of fibroblast markers (Figure S2). With pseudotime inference, we found that *FERMT2* had an essential role in the trajectory process of fibroblasts, with the evolutionary trajectories from cluster 8 to cluster 19 (Figure 3(a)). We selected 5 fibroblast activation markers including *ACTA2*, *TNC*, *ITGB1*, *CAV1*, and *FAP* to observe the changes in their expression levels during fibroblast differentiation. The results showed that the trends of them were consistent with *FERMT2*, suggesting that *FERMT2* may play a crucial role in fibroblast activation (Figures 3(b) and 3(c)). This result was validated in an independent dataset (Figure S3). We then calculated the correlation between *FERMT2* and *ACTA2* as well as *FAP* based on TCGA-STAD. The results showed that *FERMT2* maintained a strong positive correlation with *ACTA2* and *FAP* at the transcriptome level (Figure 3(d); *ACTA2*, *R* = 0.856, *P* < 0.001; *FAP*, *R* = 0.661, *P* < 0.001). To further evaluate the effect of *FERMT2* on fibroblasts, a si-*FERMT2* fibroblast cell line was established for in vitro experiments. The results showed that downregulation of *FERMT2* significantly inhibited the expression of fibroblast activation markers (Figures 3(e) and 3(f), *P* < 0.01). Histopathological sections showed histological differences between patients with different expression of *FERMT2*, and there was significant collagen accumulation in patients with high *FERMT2* expression (Figure 3(g)). Finally, we analyzed the expression of FERMT2, ACTA2, and FAP by IF staining of GC samples and confirmed their colocalization at the protein level (Figure 3(h)). Thus, *FERMT2* could be identified as a novel fibroblast marker in GC.

### 3.4. Fibroblast-Derived *FERMT2* Drives Invasive Metastasis by Facilitating EMT in GC Cells

**Figure 4:**
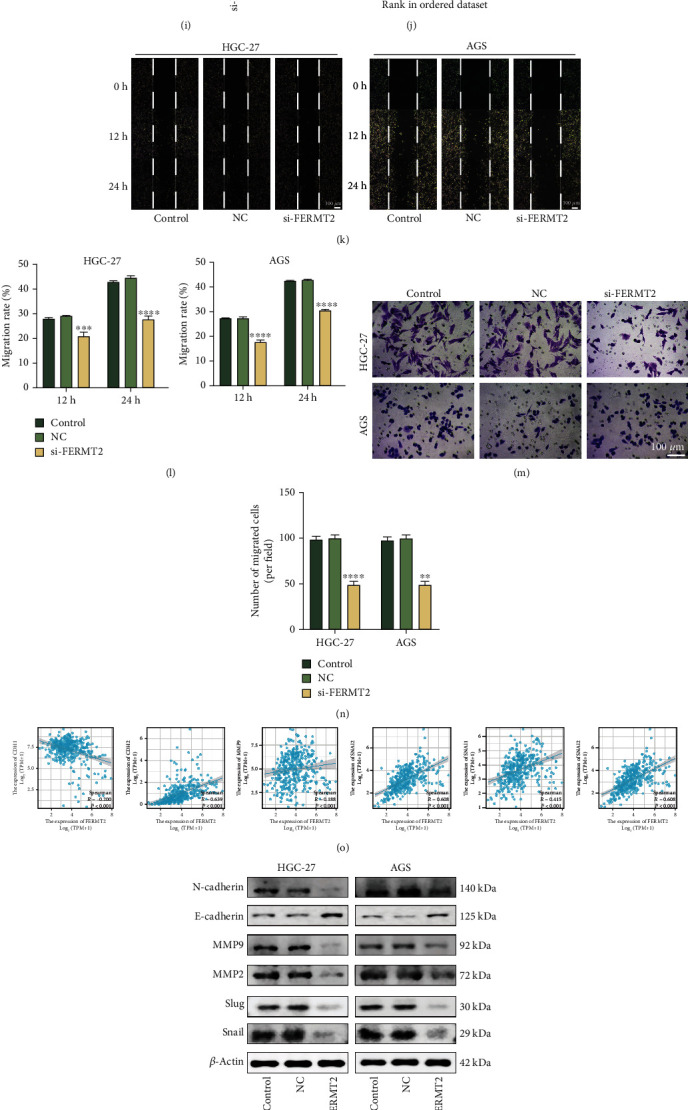
Fibroblast-derived FERMT2 promotes epithelial mesenchymal transition (EMT) in GC cells in vitro. (a) Protein-protein interaction (PPI) network for FERMT2 was constructed in GeneMANIA. Different colors of the network edge indicate the bioinformatics methods applied, including physical interaction, coexpression, and predicted. The link between FERMT2 and ZEB1 was highlighted with a red box. (b) Schematic diagram of noncontact coculture of fibroblasts and GC cells. (c, d) Western blotting results comparing FERMT2 and *β*-actin bands before and after si-FERMT2 (CAFs) (paired *t*-test). Asterisk represents *P* value (^∗∗∗∗^*P* < 0.0001). (e) The clone formation capacity of GC cells under different interventions. (f) Schematic diagram of subcutaneous tumor models. (g) Xenograft mouse tumors (*n* = 6 mice per group). (h, i) Volumes of xenograft tumors measured twice a week and weights of xenograft tumors at completion of the study. (j) Gene set enrichment analysis (GSEA) for FERMT2. The enriched gene sets in hallmark collection by the high FERMT2 expression samples. The (k, l) wound healing cell migration and (m, n) Transwell migration assays indicate that downregulation of FERMT2 in CAFs weakens the migratory and invasive capacities of GC cells (ANOVA) (scale bars = 100 *μ*m). (o) Correlation of FERMT2 expression with EMT markers in TCGA-STAD. Spearman method was used. (p, q) The expression of the EMT markers with GC cells was examined by Western blotting after the treatment of the fibroblasts with NC and si-FERMT2 constructs (ANOVA) (scale bars = 50 *μ*m). (r, s) Interfering with FERMT2 expression in vivo inhibits EMT (scale bars = 20 *μ*m). One-way ANOVA was conducted. Asterisk represents *P* value (^∗^*P* < 0.05). (t) mIHC staining images of GC section (merge and FERMT2-2×, E-cadherin and N-cadherin -20x) (scale bars = 50 *μ*m and 500 *μ*m). Each experiment was independently performed in triplicate. Each error bar indicates the variation between the means of three independent experiments. ^∗^*P* < 0.05, ^∗∗^*P* < 0.01, ^∗∗∗^*P* < 0.001, and ^∗∗∗∗^*P* < 0.0001.

EMT signal in tumor cells is at least partially driven by fibroblasts, and there is a large overlap in the gene expression profiles of mesenchymal signal and EMT, so we here further investigated the significance of *FERMT2* in control of EMT. A gene-gene interaction network was constructed using *FERMT2* as the core, and the GeneMANIA database was used to analyze the interactions of these genes. We noted that *FERMT2* was coexpressed (red box, purple lines) with *ZEB1*, which is thought to be an EMT-inducing transcription factors (Figure 4(a)). To further assess the relationship between fibroblast-derived FERMT2 and EMT in GC, a noncontact coculture model was first established (Figure 4(b)) and fibroblast cell lines were treated with *FERMT2*-specific short interfering (si) RNA (si-*FERMT2*) (Figures 4(c) and 4(d), *P* < 0.0001). In vitro experiments showed that interference with *FERMT2* expression significantly inhibited the clone formation of GC cells (Figure 4(e)). Subsequently, the si-*FERMT2* fibroblast cell line was used in an in vivo tumor growth assay (Figure 4(f)), which showed that mice injected with GC cells mixed with si-*FERMT2* fibroblasts had smaller tumor volumes than mice injected with cancer cells mixed with controls (Figures 4(g)–4(i)).

Next, we investigated the role of *FERMT2* in the regulation of EMT. GSEA further indicated a positive correlation between EMT signal levels and *FERMT2* expression (Figure 4(j)). In the above coculture model, si-*FERMT2* impaired EMT-related phenotypes represented by the migration and invasion capacity of tumor cells (Figures 4(k)–4(n), *P* < 0.05). Interestingly, *FERMT2* mRNA level was positively correlated with the mRNA level of matrix metallopeptidase 2 (*MMP2*), matrix metallopeptidase 9 (*MMP9*), snail (snail family transcriptional repressor 1 (*SNAL1*)), slug (snail family transcriptional repressor 2 (*SNAL2*)), and N-cadherin (cadherin 2 (*CDH2*)), but negatively correlated with E-cadherin (cadherin 1 (*CDH1*)) in TCGA samples (Figure 4(o), *P* < 0.05). Subsequently, the expression of these proteins was examined. The levels of MMP2, MMP9, Snail, Slug, and N-cadherin were downregulated by si-FERMT2, whereas the opposite was true for E-cadherin (Figures 4(p) and 4(q), ANOVA, *P* < 0.0001). The above in vitro findings were further confirmed in xenograft tumor in nude mice. Considerably stronger E-cadherin and lower N-cadherin staining intensities were detected in si-*FERMT2* tumor tissues relative to NC tissues. (Figures 4(r) and 4(s), *P* < 0.05). Immunofluorescence analysis of clinical tissue specimens was conducted to assess the expression patterns of FERMT2, E-cadherin, and N-cadherin in GC (Figure 4(t)). As shown in Figure 4(t), E-cadherin was barely expressed in the FERMT2 high-expression region, while N-cadherin was strongly expressed. The opposite result was found in the FERMT2 low-expression region. The results suggest that FERMT2 facilitates EMT, at least in part, through a fibroblast-dependent pathway.

### 3.5. Fibroblast-Derived FERMT2 Promotes the Growth of M2 Macrophages

**Figure 5:**
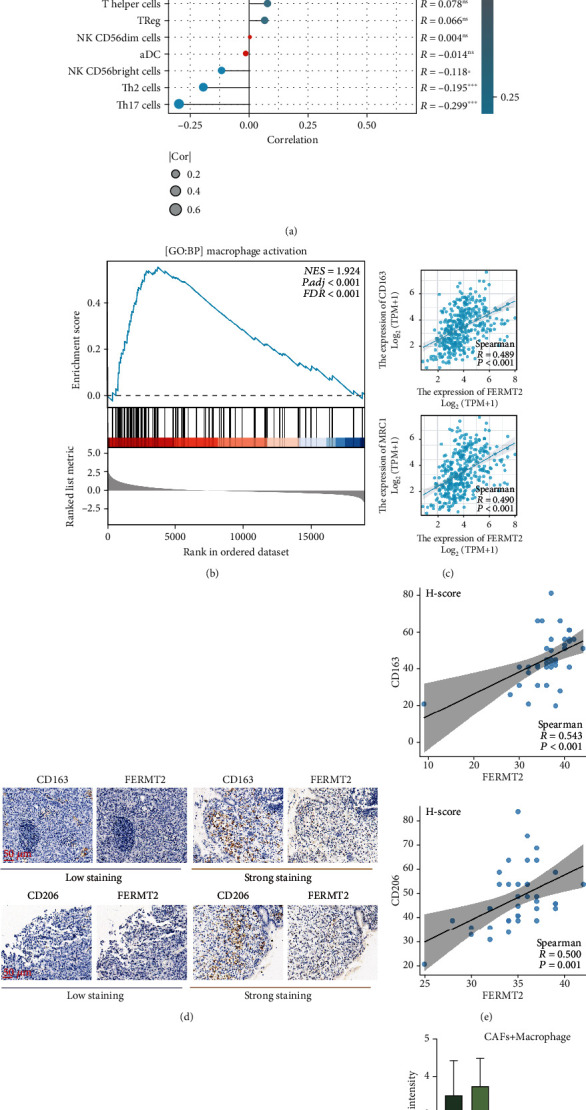
Fibroblast-derived FERMT2 promotes M2 macrophage growth in vitro. (a) The bubble chart shows the Spearman correlation between FERMT2 expression and the abundance of immune cell infiltration. The color indicates the *P* value, with red indicating smaller *P* values. The bubble size indicates the degree of correlation, with larger bubbles indicating a stronger correlation. (b) Gene set enrichment analysis (GSEA) for FERMT2. The enriched gene sets in GO collection by the high FERMT2 expression samples. (c) Correlation of FERMT2 expression with M2 macrophage markers in TCGA-STAD. Spearman method was used. (d) Immunohistochemistry analysis shows FERMT2 and the M2 macrophage markers CD163 (upper part) and CD206 (lower part) expression in serial sections of human GC tissue (scale bars = 50 *μ*m). (e) Correlation between FERMT2 and CD163 (upper part) as well as CD206 (lower part) based on immunohistochemical *H*-score calculation (Spearman method, *n* = 40). Results are representative of 3 independent experiments unless stated otherwise. (f, g) The expressions of M2 markers with macrophages were examined by immunofluorescence (IF) after the treatment of the fibroblasts with NC and si-FERMT2 constructs (ANOVA) (Scale bars = 20 *μ*m). ^∗^*P* < 0.05 and ^∗∗∗∗^*P* < 0.0001.

It is a well-known fact that macrophages and fibroblasts form a two-cell circuit through extensive cytokine exchange to promote each other [58]. Based on TCGA-STAD, we first found that the transcriptome levels of *FERMT2* positively correlated with immune markers of macrophage infiltration (Figure 5(a), *R* = 0.485, *P* < 0.001). GSEA indicated that *FERMT2* may be involved in macrophage activation (Figure 5(b), normalized enrichment score = 1.924, FDR < 0.001). Further calculations showed that FERMT2 was significantly and positively correlated with the markers of M2-like macrophages in Figure 5(c) (CD163, *R* = 0.489, *P* < 0.001; mannose receptor C-type 1 (MRC1), *R* = 0.490, *P* < 0.001) levels. In addition to data at the transcriptional level, IHC staining confirmed a decrease in protein levels of CD163 and CD206 (representing tumor-infiltrating M2 macrophages) with increased level of FERMT2 proteins (Figures 5(d) and 5(e); CD163, *R* = 0.543, *P* < 0.001; CD206, *R* = 0.500, *P* = 0.001). Finally, in the coculture system described above, we confirmed that intervention of *FERMT2* expression in fibroblasts significantly weakened the ability of fibroblasts to activate M2 macrophages (Figures 5(f) and 5(g), *P* < 0.05). In conclusion, these results suggest that *FERMT2* may be potential biological markers of TME alterations, especially in terms of immunosuppression.

### 3.6. FERMT2 Associates with Resistance to Immunotherapy in GC

**Figure 6:**
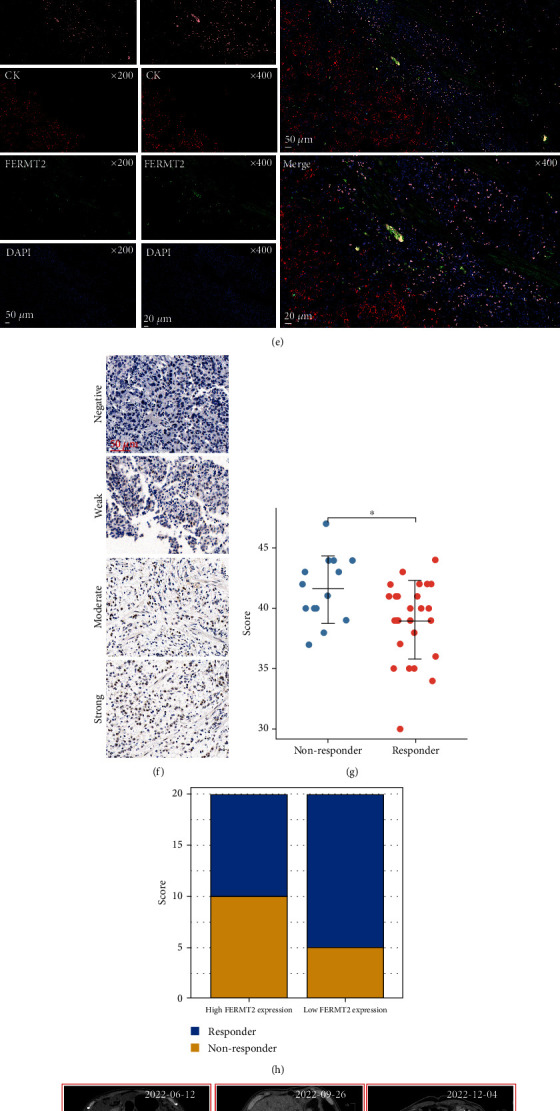
FERMT2 is a detrimental factor for immunotherapy in GC patients. (a) Correlation between *FERMT2* expression and TIDE score as analyzed based on TCGA-STAD (Spearman method, *n* = 375). (b) Expression correlation analysis of *FERMT2* and programmed cell death 1 (*PDCD1*) based on TCGA-STAD (Spearman method, *n* = 375). (c) Immunohistochemistry for FERMT2 and PDCD1 was performed on GC serial sections (*n* = 40). (d) Statistical comparison of FERMT2 and PDCD1 expression levels (*H*-score) was analyzed using the Spearman correlation analysis (*n* = 40). (e) Representative costained images of FERMT2, CK, and CD8 in the immune excluded immunophenotypes. Scale bars, 100 *μ*m and 20 *μ*m enlarged images. Nuclei (DAPI) in blue. (f) Representative images of different immunohistochemical staining intensities for FERMT2 based on our own GC samples (*n* = 40). (g) Box plot showing distinct FERMT2 expression (*H*-score) between responder and nonresponder after anti-PD-1 therapy in 40 GC patients (Wilcoxon test, ^∗^*P* < 0.05). (h) Bar plot showing distinct response rates between the high- and low-FERMT2 groups in 40 GC patients. Representative pictures of CT (computed tomography) scan of FERMT2 (i) high-expression and (j) low-expression GC patients. The expression level of FERMT2 can predict the efficacy of immunotherapy. The arrows indicate the primary or metastatic tumor foci. Red for progressive disease (PD), green for partial response (PR), and blue for stable disease (SD).

Frequent interactions between macrophages and fibroblasts help tumor cells to complete immune escape and establish an immunosuppressive microenvironment, which is a major obstacle to immunotherapy [59, 60]. Considering that immunotherapy is the standard first-line systemic therapy for patients with GC, we calculated the correlation between immunotherapy response rates and *FERMT2* by TIDE score, and the results showed there was a strong correlation between *FERMT2* level and TIDE score (Figure 6(a), *R* = 0.488, *P* < 0.001). Because the TIDE score takes into account factors including immune checkpoint levels and immunosuppressive cell abundance, we first calculated the correlation between *FERMT2* and (programmed cell death 1 (*PD-1*)) and found that the correlation between them was very weak at the transcriptome level (Figure 6(b); TCGA-STAD; *R* = 0.163, *P* = 0.002) and at the protein level (Figures 6(c) and 6(d); our samples; *R* = 0.208 for FERMT2 and PD-1, *P* = 0.197). Subsequently, we found in IF staining of GC samples that FERMT2 could form a barrier to prevent CD8+ T cells from entering the tumor tissue to exert their killing effect (Figure 6(e)). Finally, we retrospectively collected 40 GC patients and assigned them FERMT2 levels by IHC (Figure 6(f)), showing that patients who responded to immunotherapy had lower FERMT2 levels than nonresponders (Figure 6(g), *P* < 0.05) and that patients with high FERMT2 expression presented lower response rates compared to those with low FERMT2 expression (Figure 6(h)). The imaging data showed that patients with high FERMT2 expression (arrows represent metastatic foci) had earlier disease progression compared to the group with low expression (Figures 6(i) and 6(j)). This validates our previous deduction that FERMT2 is a detrimental factor that can impact the outcome of immunotherapy. Therefore, *FERMT2* is a potential immune exclusion factor.

## 4. Discussion

As one of the most lethal malignancies, knowledge about GC heterogeneity remains scarce [61]. Like most focal adhesion proteins, fermitin family homolog-2 (FERMT2), localized at the site where the extracellular matrix (ECM) connects to the actin cytoskeleton, is involved in essential extracellular matrix-based tasks such as migration, proliferation, and differentiation [62–64]. FERMT2 has been found to interact with integrins to regulate many physiopathological processes; most notably, FERMT2 has been identified as being associated with amyloid precursor protein (APP) metabolism, which is a significant risk factor for Alzheimer's disease (AD) [65]. On the other hand, *FERMT2* has been identified as a novel oncogene that is highly expressed tumors, including breast, pancreatic, and colorectal cancers, and promotes invasion and metastasis [34, 36, 66, 67].

GC contains a rich tumor microenvironment in which nontransformed components (particularly fibroblasts and macrophages) are thought to be important components of the tumor supporting microenvironment [68, 69]. Transforming growth factor beta 2 (TGF*β*2) secreted by tumor-associated macrophages (TAMs) promotes GC cell invasion by promoting the level of FERMT2 [70]. In this study, we collected forty surgical resection samples and determined that FERMT2 was highly enriched in fibroblasts using mIF staining. In addition, using the ESTIMATE tool to calculate the proportion of stromal components in pancancerous tissues, *FERMT2* was found to be an indicator of high stromal abundance. Mature fibroblasts have the ability to increase ECM deposition, specifically by creating a matrix rich in fibronectin and collagen to protect tumor cells from infiltrating lymphocytes [71]. Our study revealed that *FERMT2* promotes fibroblast activation in the GC mesenchyme and is a poor prognostic indicator. Importantly, in conditions characterized by pathological tissue fibrosis (e.g., pancreatic cancer), fibroblasts sense the stiffened ECM by establishing a mechanical feedback loop, thereby contributing to the perpetuation of their fully activated state [72]. ECM sclerosis has been shown to promote FERMT2 translocation to mitochondria and its interaction with pyrroline-5-carboxylate reductase 1 (PYCR1), which regulates crosstalk between the tumor and its microenvironment in breast cancer [73]. We hypothesized that there may be a positive feedback mechanism between FERMT2 and that it may serve as an important component of the fibroblast-stroma feedback loop.

Previous studies have shown that stromal cells emitting pro-EMT signals help tumor cells acquire the EMT phenotype more readily [74]. Gene-gene interaction network analysis implied a close association between *FERMT2* and the EMT transcription factor *ZEB1*. Experiments confirmed that *FERMT2* promotes EMT in GC cells in a fibroblast-dependent manner, thereby driving invasion and metastasis. Interestingly, in the bulk data, we observed significantly high expression of *FERMT2* in advanced and poorly differentiated GC. Advanced disease is characterized by fibroblast-driven fibrosis [75], and in parallel, poorly differentiated tumors generally undergo a more complete EMT process [76]. We speculated that *FERMT2* may be a key factor in the progression of GC, particularly with respect to the mesenchymal signal.

With the help of ssGSEA, we discovered a close link between *FERMT2* and macrophages. Further calculations showed that *FERMT2* positively correlated with the marker levels of M2-like macrophages. It is well known that macrophage signaling pathways are severely upregulated by fibroblasts [77, 78] and that the latter also thereby mediate the polarization of macrophages towards the M2 subtype [79]. Our study demonstrated that fibroblast-derived *FERMT2* promoted the growth of M2 macrophages. The high level of M2 macrophage infiltration in gastric cancer TME is an unfavorable prognostic factor, which is consistent with the negative effect of *FERMT2* on survival. In addition, IHC of clinical specimens provided evidence for the clinical use of FERMT2 and M2 macrophage markers in prognostic assessment.

To date, the role of *FERMT2*, particularly its role in immune response and immune escape, remains unclear in GC. High *FERMT2* expression positively correlated with the abundance of nonparenchymal cells in gastric cancer TME, which limited the efficacy of immunotherapy by shaping the immunosuppressive microenvironment. In melanoma, the expression level of *FERMT2* was identified to be associated with the efficacy of target therapies [80]. Although *FERMT2* is associated with the clinical features of GC patients and has been shown to be an upstream factor promoting GC invasion, the gene was found to have a low correlation with the currently well-known immune checkpoint PD-1. Combined with previous results, we hypothesized that the role of *FERMT2* was independent of well-known immunomodulatory mechanisms and was more biased towards stromal signal aspects. Subsequently, in a small clinical cohort, we found that patients with high *FERMT2* expression had more difficulty benefiting from immunotherapy. More interestingly, IF showed that FERMT2 was able to form a restrictive physical barrier, preventing CD8+ T cells from killing tumor cells.

We acknowledge that there are some limitations to the present study. First, similar to all bioinformatics-based algorithms for inferring the composition of tumor infiltration, our calculations may have ignored cell types that were not assessed by the applied algorithm but are significant for TME. Second, since the strength of mesenchymal signaling differs between the core region of the tumor entity and the infiltrative margin, assessing intertumoral heterogeneity accordingly would be more helpful in elucidating the complex function of *FERMT2*. Third, the number of included clinical samples was small, and it was necessary to collect patients in multicenter clinical queues for further analysis and validation.

## 5. Conclusion

Our study shows that *FERMT2* is a high-abundance stroma-associated gene with prognostic signaling associated with M2 macrophages, further contributing to EMT labeling. The combination of the fibroblast/FERMT2/EMT/M2 macrophage axis may affect the prognosis and response to immunotherapy in GC patients. Signal crosstalk between the FERMT2-expressing tumor stroma and GC cells is of interest. Further studies with this goal will help provide new insights into target therapies and immunotherapy for GC.

## Data Availability

We declare that all the data in this article are authentic, valid, and available for use on reasonable request.

## Supplementary Materials

Supplementary Materials The details for all antibodies, reagents and other details, and table and supplementary figures are provided in the supplementary materials.
