# Supplementary material for: Overexpression of FERM Domain Containing Kindlin 2 (FERMT2) in Fibroblasts Correlates with EMT and Immunosuppression in Gastric Cancer
Source: Int J Genomics. 2024 Feb 6;2024:4123737. doi: 10.1155/2024/4123737 (PMC10864055; doi:10.1155/2024/4123737)
Supplement: Supplementary Materials — The details for all antibodies, reagents and other details, and table and supplementary figures are provided in the supplementary materials. [file 4123737.f1.docx]

Supplementary material

# Supplementary Table

## Table S1

| Antibodies and Reagents | Manufacturer, Country, Cat number | Concentration |
| --- | --- | --- |
| HE staining kit | Beyotime Biotechnology, China, #C0105, | - |
| Goat Anti-Rabbit IgG H&L (HRP) | Abcam, UK, #ab205718 | IHC 1:2000 |
| Alexa Fluor 488 AffiniPure goat anti-mouse IgG (H+L) | FcMACS, China, #136908 | IF1:500 |
| Goat anti-mouse IgG (H+L) CoraLite594 | Proteintech, China, #SA00013-4 | IF 1:500 |
| Fluorescein (FITC) conjugated Affinipure Goat Anti-Rabbit IgG(H+L) | Proteintech, China, # SA00003-2 | IF 1:500 |
| Plasmid vectors for *FERMT2* | GeneChem, China, #10021850 |  |
| Puromycin | Beyotime Biotechnology, China, # 041321210517 |  |
| Lipofectamine 3000 | Invitrogen, USA, #2395298 |  |
| 0.2% TritonX-100 | Proteintech, China, #61202011 |  |
| Nuclei were stained using 4',6-diamidino-2-phenylindole (DAPI) | Beyotime Biotechnology, China, # 091620210520 |  |
| phorbol 12-myristate 13-acetate (PMA) | Sigma-Aldrich, USA, #63597-44-4 | 10 ng/mL |
| anti-CD163 polyclonal antibodies | Proteintech, China, # [HPA046404](https://www.sigmaaldrich.cn/CN/zh/product/sigma/hpa046404) | 1:1000 |
| anti-CD206 polyclonal antibodies | Proteintech, China, # [SAB5700929](https://www.sigmaaldrich.cn/CN/zh/product/sigma/sab5700929) | 1:1000 |
| Penicillin/streptomycin | Gibco, USA, # 15070063 |  |
| Anti-β-actin Antibody | Invitrogen, USA, # PA5-21396 | WB: 1:5000 |
| Anti-E-cadherin Antibody | Cell Signaling Technology, USA.# [96743](https://www.cellsignal.cn/products/primary-antibodies/e-cadherin-24e10-rabbit-mab-bsa-and-azide-free/96743?site-search-type=Products&N=4294956287&Ntt=anti-e-cadherin+antibody&fromPage=plp) | WB: 1:1000  IF: 100 |
| Anti-N-cadherin Antibody | Cell Signaling Technology, USA. # [4061](https://www.cellsignal.cn/products/primary-antibodies/n-cadherin-antibody/4061?site-search-type=Products&N=4294956287&Ntt=anti-n-cadherin+antibody&fromPage=plp) | WB: 1:1000  IF: 100 |
| Anti-MMP2 Antibody | Cell Signaling Technology, USA. # [4022](https://www.cellsignal.cn/products/primary-antibodies/mmp-2-antibody/4022?site-search-type=Products&N=4294956287&Ntt=anti-mmp2+antibody&fromPage=plp) | WB: 1:1000 |
| Anti-MMP9 Antibody | Cell Signaling Technology, USA. # [3852](https://www.cellsignal.cn/products/primary-antibodies/mmp-9-antibody/3852?site-search-type=Products&N=4294956287&Ntt=anti-mmp9+antibody&fromPage=plp) | WB: 1:1000 |
| Anti-Snail Antibody | Invitrogen, USA, # PA5-23482 | WB: 1:1000 |
| SLUG Polyclonal Antibody | Invitrogen, USA, # PA5-20289 | WB: 1:1000 |
| FAP Antibody for Immunofluorescence | Cell Signaling Technology, USA. # [66562](https://www.cellsignal.cn/products/primary-antibodies/fap-e1v9v-rabbit-mab/66562?site-search-type=Products&N=4294956287&Ntt=fap&fromPage=plp) | IF: 1:500 |
| α-SMA Antibody for Immunofluorescence | Cell Signaling Technology, # [19245](https://www.cellsignal.cn/products/primary-antibodies/a-smooth-muscle-actin-d4k9n-xp-rabbit-mab/19245?site-search-type=Products&N=4294956287&Ntt=%CE%B1-sma&fromPage=plp) | IF: 1:500 |
| CD8 Polyclonal Antibody | Thermo Fisher Scientific, US, # PA5-83066 | IF 1:200 |
| Highly Cross-Adsorbed Goat (Polyclonal) Anti-Mouse IgG(H+L) Antibody | LI-COR, USA, #926-68070 | WB 1:5000 |
| Highly Cross-Adsorbed Goat (Polyclonal) Anti-Rabbit IgG(H+L) Antibody | LI-COR, USA, #926-68071 | WB 1:5000 |
| Dulbecco’s modified Eagle’s medium (DMEM) | Gibco, USA, # 11965092 | - |
| Fetal bovine serum (FBS) | Gibco, USA, # 10091148 | - |
| Roswell Park Memorial Institute 1640 (RPMI-1640) | Gibco, USA, # 11875119 | - |
| Kindlin 2 Monoclonal Antibody (FERMT2) | Thermo Fisher Scientific, US, # MA5-24937 | IHC 1:200  IF 1:100 |
| PD-1 Monoclonal Antibody | Thermo Fisher Scientific, US, # MA5-15780 | IHC 1:200 |
| Cytokeratin 20 Recombinant Rabbit Monoclonal Antibody (CK) | Thermo Fisher Scientific, US, # MA5-31979 | IF 1:200 |

# Supplementary Figures

## Figure S1.


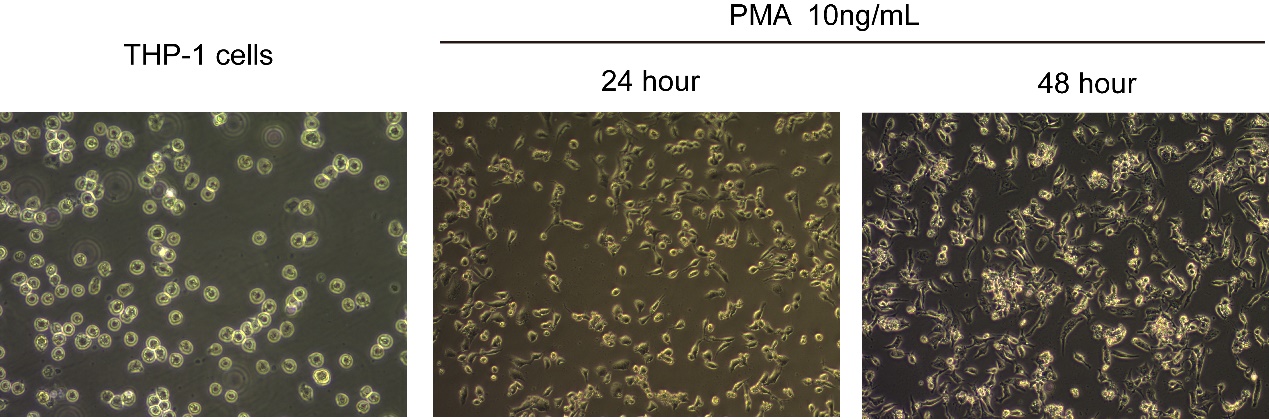


Morphological change of M0 macrophages derived from THP-1 monocytes. It was studied under the Olympus BX50 light microscope (200× magnification)

## Figure S2.


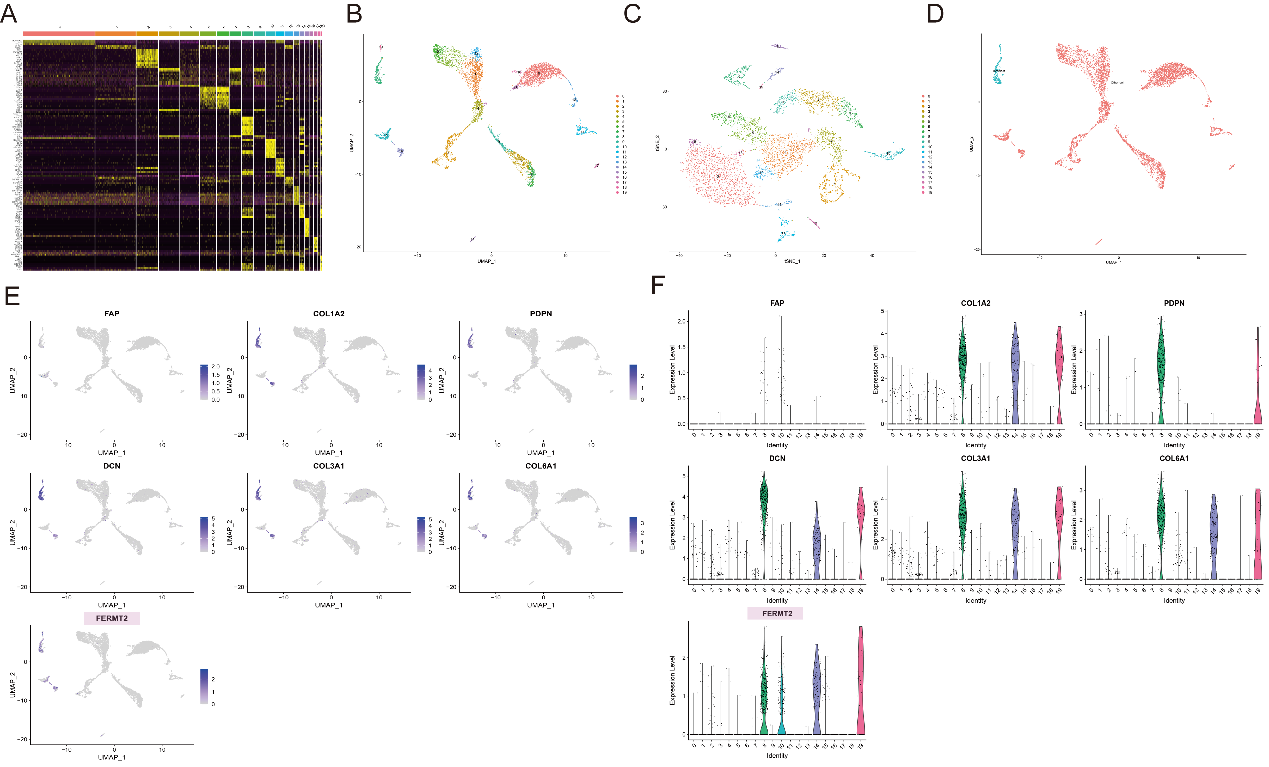


Single cell analysis of *FERMT2*.

(A) Cell-type markers. The relative expression level of genes across cells is shown, sorted by cell type. Cell-type marker genes were identified in an unbiased fashion (Wilcoxon rank-sum test, FDR <0.01, and fold change >1.5) and only the top 15 gene are shown in the figure.

(B-C) Cells were clustered into 19 types via Uniform Manifold Approximation and Projection (UMAP) (B) and t-Distributed Stochastic Neighbor Embedding (t-SNE) (C) plot dimensionality reduction algorithm, each color represents a unique cluster.

(D) Fibroblasts are distinguished from all other cells based on UMAP dimensionality reduction analysis.

(E-F) Dot plots (E) and violin plots (F) demonstrate the expression distribution of fibroblast activation marker genes and *FERMT2*.

## Figure S3.


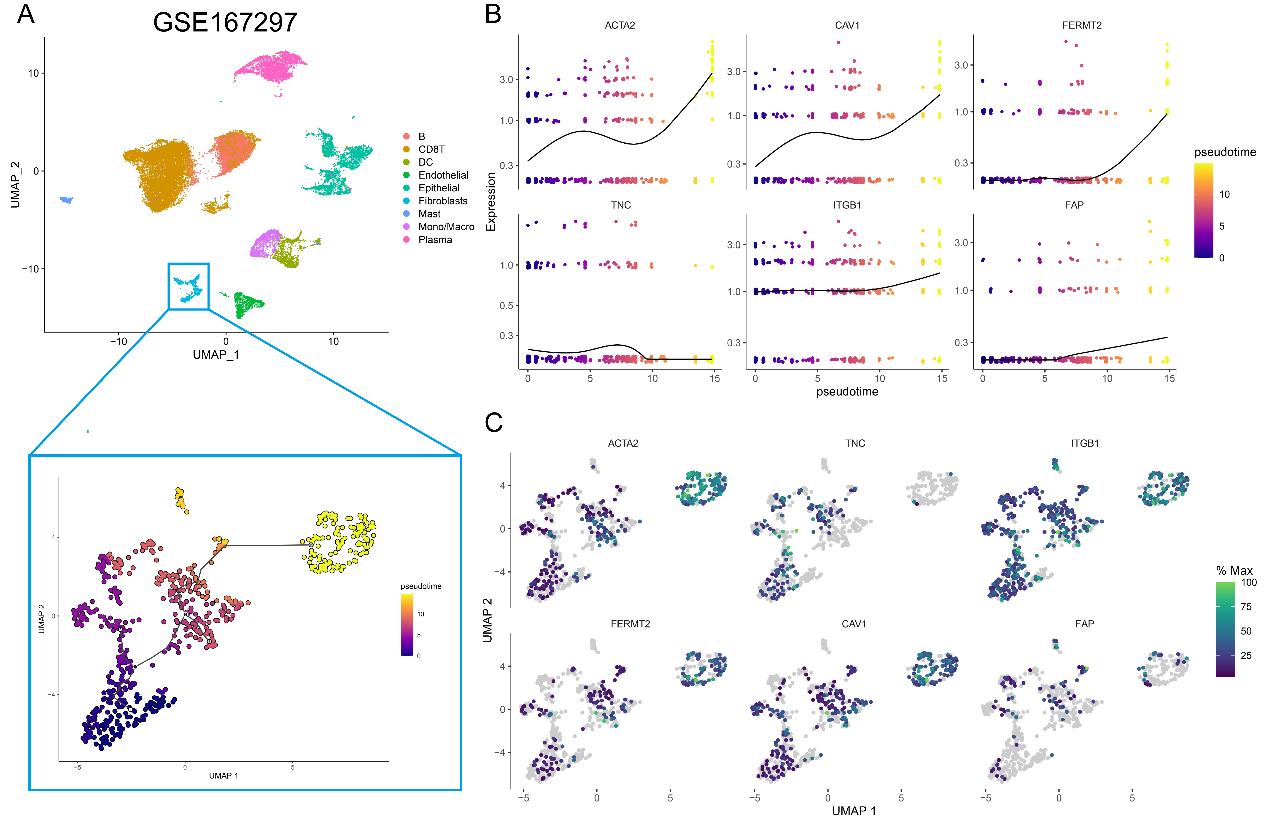


Pseudotime-ordered analysis of fibroblasts from samples in GSE167297.

(A) Pseudotime trajectory of all the fibroblasts from cluster 1 to cluster 3. All the fibroblasts were colored by their assigned pseudotime values.

(B) Jitter plots showing the expression level of the fibroblast activation markers and FERMT2 changing with pseudotime.

(C) UMAP dimensionality reduction visualizes similarity of expression profiles of FERMT2 and fibroblast activation markers.
